# Comparative Evaluation of Staining Techniques in Thawed Cryopreserved Dog Semen

**DOI:** 10.3390/vetsci13070627

**Published:** 2026-06-27

**Authors:** Indra Sara Klumb, Axel Wehrend, Abbas Farshad

**Affiliations:** Veterinary Clinic for Reproductive Medicine and Neonatology, Justus-Liebig-University of Giessen, 35392 Giessen, Germanyaxel.wehrend@vetmed.uni-giessen.de (A.W.)

**Keywords:** dog, cryopreservation, spermatozoa, staining method, light microscopy

## Abstract

Background: Accurate assessment of sperm morphology is essential for evaluating the quality of cryopreserved canine semen used in artificial insemination and for improving cryopreservation protocols. This study compared six staining techniques, Eosin, Eosin–Nigrosin, Diff-Quick^®^, Hemacolor^®^, Spermac^®^, and Formol-citrate Bengal Rose, for light-microscopic evaluation of frozen–thawed canine spermatozoa. Methods: Semen from ten dogs was thawed, divided into four aliquots, and either left untreated or exposed to thermal stress at 6 °C, 18 °C, or 37 °C for two hours to induce morphological variation. A total of 360 slides and 960 evaluations were performed immediately after staining and again after 24 h, 7 days, and 3 months to assess staining quality and stability over time. Results: Eosin produced stable staining for up to three months and was the most economical method, though its initial detail recognition was lower than that of Spermac^®^ and Formol-citrate Bengal Rose. Eosin–Nigrosin showed reduced contrast and detail. Diff-Quick^®^ provided better contrast than Eosin–Nigrosin, while Hemacolor^®^ maintained consistent quality regardless of stress treatment or storage duration. Spermac^®^ yielded the highest initial morphological detail but deteriorated during storage. Formol-citrate Bengal Rose combined high detail recognition with stable staining throughout the study. Cryopreservation increased looped tails, and incubation at 37 °C markedly elevated pathological sperm and rudimentary tails. Conclusions: All six staining methods were suitable for evaluating and archiving frozen–thawed canine semen. Formol-citrate Bengal Rose and Spermac^®^ offered the best detail, while Eosin provided a cost-effective option with excellent long-term stability.

## Simple Summary

Accurate sperm morphology assessment is important for evaluating frozen canine semen used in artificial insemination. This study compared six staining methods for examining frozen–thawed dog sperm and assessed their performance immediately after staining and during storage for up to three months. All methods were suitable for routine evaluation and long-term slide archiving. Spermac^®^ provided the greatest morphological detail, while Formol-citrate Bengal Rose combined high detail recognition with stable staining quality over time. Hemacolor^®^ showed consistent performance, and Eosin was the most economical option. Cryopreservation increased looped sperm tails, and exposure to 37 °C increased the proportion of abnormal sperm. These results provide practical guidance for selecting staining methods for canine semen evaluation.

## 1. Introduction

The light microscopic examination of stained sperm cell preparations is an essential component of spermatological diagnostics and plays a central role in the assessment of semen quality. In the dog, sperm morphology is considered a major determinant of fertilizing capacity, and an increased proportion of morphologically abnormal spermatozoa has been associated with reduced fertility [1]. Depending on the reference, proportions of 10–25% [2] or 20–30% [3] morphologically abnormal spermatozoa are regarded as physiological in canine semen. Together with total sperm count and sperm viability, sperm morphology represents one of the three most important parameters for semen quality assessment in the dog [4]. Light microscopic morphological evaluation is routinely included in spermatological examinations of canine semen and has been applied in numerous experimental and clinical studies [5,6,7,8,9]. The importance of sperm morphology as a diagnostic parameter has also been emphasized in human medicine, where it has been described as one of the most reliable criteria for distinguishing fertile from infertile males [10].

A wide variety of staining techniques are available for the light microscopic evaluation of spermatozoa. These methods differ in their ability to visualize sperm structures such as the head, acrosome, midpiece, and tail, as well as in their suitability for assessing sperm viability, membrane integrity, and pathomorphological alterations. Despite their routine use in veterinary andrological diagnostics, many of these staining methods have not been systematically compared, particularly regarding canine semen and cryopreserved samples. Among the most frequently applied stains are Eosin-based techniques. Eosin is a vital dye that penetrates spermatozoa with damaged plasma membranes, thereby allowing differentiation between viable and non-viable cells [11]. In addition to vitality assessment, Eosin-stained preparations can also be used for morphological evaluation [12]. Reported normative values for Eosin-positive spermatozoa in dogs range from 5 to 10%, although higher proportions of non-viable spermatozoa may still be considered within physiological limits [12,13].

The combination of Eosin with Nigrosin enhances contrast by creating a dark background, facilitating both vitality assessment and morphological evaluation [2]. The Eosin–Nigrosin staining technique has been widely applied in numerous animal species and is regarded as a standard method for routine sperm evaluation in the dog [4,13,14]. Several studies have demonstrated its suitability for highlighting morphological abnormalities and differentiating live from dead spermatozoa, although results may be influenced by staining duration, temperature, and storage conditions [15,16,17]. Commercial rapid staining methods such as Diff-Quick^®^ and Hemacolor^®^ are also commonly used in spermatological diagnostics. Diff-Quick^®^, a Romanowsky-type stain, allows rapid preparation and provides good contrast of sperm structures, including the acrosome and postacrosomal region [18]. Comparative studies in human and animal sperm have shown that Diff-Quick^®^ yields result comparable to more time-consuming staining methods while offering advantages in terms of speed and standardization [19,20]. In canine semen, Diff-Quick^®^ has been shown to produce a high proportion of morphologically normal spermatozoa and is currently considered a standard staining technique for the assessment of sperm pathomorphology [4].

Hemacolor^®^, a rapid staining method based on the May–Grünwald–Giemsa technique, has primarily been used in hematology but has also been applied to sperm evaluation. While some studies reported reduced contrast compared to other stains [21], others found no significant differences in staining quality when applied to animal spermatozoa [22]. Hemacolor^®^ has been shown to be suitable for the assessment of sperm head morphometry in certain species [23] and may be useful in specific diagnostic contexts, such as the detection of inflammatory cells in canine semen [24]. For the selective assessment of acrosomal integrity, Spermac^®^ is widely used. This multistep staining technique enables clear differentiation between intact and damaged acrosomes and has been successfully applied in canine and human semen analysis [25,26]. Spermac^®^ is regarded as particularly suitable for the evaluation of cryopreserved semen, as freezing and thawing processes frequently induce acrosomal damage [4,27]. The method has demonstrated good reliability, contrast, and detail recognition in boar [22].

Another method used for morphological and acrosomal assessment is Formol-citrate Bengal Rose staining. Bengal Rose stains acrosomal structures uniformly and allows identification of acrosomal defects based on changes in shape and staining intensity [28]. Although the method does not provide selective acrosomal staining, it has been successfully applied in various species, including the dog and boar, and offers good color intensity and detail recognition [22,26]. In addition to these established staining techniques, numerous other methods have been described for canine sperm evaluation, including bromophenol-based stains, methyl violet, Papanicolaou, toluidine blue, aniline blue, and protein-specific dyes such as Coomassie blue [4,28,29,30]. Fluorescence-based techniques and flow cytometry allow highly sensitive and objective assessment of sperm viability, membrane integrity, acrosomal status, and chromatin condensation [31,32]. However, due to high costs and specialized equipment requirements, these methods are not routinely available in veterinary practice. An important factor influencing the evaluation of sperm morphology is storage duration and sample processing. This is particularly relevant for cryopreserved semen and for retained samples used for quality control or forensic purposes, as delayed examination may alter staining behavior and morphological appearance over time [17,22,33].

Beyond these methodological considerations, accurate morphological evaluation is particularly important because spermatozoa are among the most morphologically diverse cell types in the animal kingdom, reflecting strong evolutionary and functional constraints [34,35,36]. At the same time, sperm morphometry is highly sensitive to laboratory processing, especially fixation and staining procedures, which may introduce artefacts and alter measured dimensions in a method-dependent manner [22,37]. Consequently, results obtained using different staining protocols are not always directly comparable across studies, species, or laboratories, highlighting the importance of standardization and species-specific validation [35,38]. Despite the widespread use of staining techniques for semen evaluation, no systematic comparison of staining methods has been performed for canine spermatozoa, particularly in thawed, cryopreserved samples that are highly relevant in clinical reproduction, breeding programs, and forensic investigations. Furthermore, staining methods differ in their ability to visualize sperm structures and detect morphological abnormalities, which may influence diagnostic interpretation [22,28,37]. Their performance may also be affected by storage duration, an important consideration when archived or retained samples are examined after prolonged periods [17,22,33]. Therefore, the aim of the present study was to compare six staining methods for the evaluation of thawed, cryopreserved canine semen, focusing on their diagnostic suitability, the influence of storage time on sample evaluability, and practical aspects including processing time and cost.

## 2. Materials and Methods

### 2.1. Experimental Design and Procedures

This study used thawed semen from ten fertile, privately owned dogs presented to the Clinic for Reproductive Medicine and Neonatology in Giessen for routine semen evaluation and cryopreservation. The study population comprised different breeds, including Border Collie (*n* = 2), Rhodesian Ridgeback (*n* = 2), Mastiff, American Bulldog, Briard, German Shepherd Dog, Eurasier, and American Akita (each *n* = 1). All dogs were clinically healthy at the time of collection, exhibited normal libido, and showed no abnormalities on general or reproductive examination. The dogs had an average age of approximately four years. Ejaculates were obtained by manual stimulation following established procedures [39]. The sperm samples were diluted in the clinic laboratory using the Uppsala Equex-2 system (CaniRep HB, Uppsala, Sweden). After an initial cooling phase of 1 h at refrigerator temperature, followed by an additional 15 min at 4 °C over nitrogen vapor, the insemination straws were deep-frozen and stored in a liquid-nitrogen container at −196 °C [33]. For experimental analysis, two 0.5 mL straws per dog were thawed at 37 °C for five minutes according to WHO guidelines [40]. Thawed semen was pooled and divided into four aliquots (0.25 mL each). One aliquot was analyzed immediately, whereas the remaining three were subjected to a two-hour incubation at 6 °C, 18 °C, or 37 °C to induce morphological alterations. Incubation at 18 °C was carried out at room temperature under continuous thermometer monitoring. Samples at 37 °C were maintained in microcentrifuge tubes on a temperature-controlled heating plate (Minitüb, model HT 200, Minitüb GmbH, Tiefenbach, Germany), ensuring stable conditions, while those at 6 °C were stored in a refrigerator with continuous monitoring using an alarm-equipped laboratory thermometer (TFA, Lab Thermometer IP65 LT-102, TFA Dostmann GmbH & Co. KG, Wertheim, Germany). Immediately after incubation, all samples were processed for smear preparation, staining, and subsequent morphological evaluation.

In each stained sample, 200 spermatozoa were evaluated for viability (Eosin and Eosin (Art. Nr. 15405/0025, Minitüb GmbH, Darmstadt, Germany)–Nigrosin, Art. Nr. 15405/0029, Minitüb GmbH, Tiefenbach, Germany) and morphological abnormalities, and relative percentages were calculated. Slides were examined in a meandering pattern using an electronic cell counter (Assistent® AC-15; Karl Hecht GmbH & Co KG, Sondheim/Rhön, Germany). Microscopic assessments were performed at 400× Eosin, Diff-Quick^®^ (Labor + Technik Eberhard Lehmann GmbH, Art. No. LT 001 and 1210, Berlin, German) and Hemacolor^®^ (Art. Nr. 1.11674.0001, Merck KGaA, Darmstadt, Germany) or 1000× magnification with oil immersion (Eosin–Nigrosin, Spermac^®^, Formol-citrate Bengal Rose) using a microscope (Hund H500, Wetzlar, Germany). Pathomorphological evaluation followed the WHO laboratory manual for the examination and processing of human semen [40], adapted to canine sperm morphology based on established references [4,22], and considered the normal structural organization of the spermatozoon into head, midpiece, and tail.

To assess storage stability, slides were coverslipped after the initial evaluation using Entellan^®^ Neu (Nr. 1.07961.0500, Fa. Merck KGaA, Darmstadt, Germany) in accordance with WHO [40] recommendations and dried horizontally for 30 min. Wet preparations stained with Formol-citrate Bengal Rose were not additionally sealed. Slides were stored dry, at room temperature, and protected from light until re-evaluation. Repeated examinations were conducted immediately after preparation (t_0_), after 24 h (t_1_), after 7 days (t_2_), and after 3 months (t_3_). Pathomorphological parameters were reassessed and compared with baseline values to evaluate long-term stability and suitability for archiving. The entire analysis comprised 160 evaluation procedures per staining method, corresponding to the four examination time points. Across the six staining methods, this resulted in 960 assessments performed on the prepared slides. Results were initially recorded manually and subsequently transferred to Excel^®^. Preparation time and costs per slide were calculated based on consumable materials and labor costs of a veterinary technician. Standard laboratory equipment was excluded. Preparation and evaluation times were recorded once at t_0_ for the first ten samples, assuming pre-thawed semen and ready-to-use reagents. Both slide preparation and evaluation of 200 spermatozoa were timed to support method selection under routine clinical conditions.

### 2.2. Staining Methods

Six unique staining methods were utilized to examine the specific morphological characteristics of cryopreserved canine spermatozoa. The protocols were executed either on the manufacturer’s guidelines or established references from the literature [41]. Due to differences in their fundamental principles, these stains vary in terms of target structures, preparation techniques, and diagnostic purposes. To enhance the morphological evaluation, the quality of staining was quantitatively assessed using three criteria: color intensity, detail recognition, and contrast. Each criterion was scored on a 7-point scale (1 = lowest, 7 = highest) to allow standardized comparison across all methods. Each slide was evaluated to generate a robust dataset with a sufficient sample size, ensuring consistency and enabling comparison across staining techniques.

#### 2.2.1. Eosin and Eosin–Nigrosin Staining

A 2% eosin solution (Art. No. 15405/0025, Minitüb GmbH, Tiefenbach, Germany) was prepared by dissolving 2 g of Eosin B and 3 g of trisodium citrate dihydrate in 100 mL of distilled water. For each smear, 10 µL of semen was mixed with 10 µL of the eosin solution on a prewarmed slide, spread with a second slide held at 45°, and air-dried. Slides were examined at 400× magnification. Dead sperm absorbed the dye and appeared pink to red, while live sperm remained unstained, allowing viability to be expressed as the percentage of stained cells [42,43]. Sperm evaluation was performed at 400× magnification using a light microscope.

Eosin–Nigrosin staining (Eosin-Art. No. 15405/0025, Nigrosin-Art. No. 15405/0029, Minitüb GmbH, Tiefenbach, Germany) allows simultaneous assessment of sperm viability and morphology. Equal volumes of semen and stain (10 µL each) were mixed on a slide. Sperm evaluation was performed at 1000× magnification using oil immersion on a light microscope. Two slides were prepared to ensure that, for repeat examinations, areas not previously exposed to immersion oil were available. Live sperm appeared pale against a dark background, while dead cells were stained pink to red [42,43].

#### 2.2.2. Diff-Quick^®^ and Hemacolor^®^ Staining

Diff-Quick^®^ staining was performed on pre-prepared, air-dried smears using the LT-SYS^®^ rapid staining kit (Art. No. LT 001 + 1210, Labor + Technik Eberhard Lehmann GmbH, Berlin, Germany). The fixative solution (methanol, methylene blue) first immobilized the sperm on the slide. This was followed by immersion in Solution I, containing phosphate buffer, eosin, and detergents, which stained basic cell components red. Solution II, composed of phosphate buffer and azure, then stained acidic structures blue. Excess stains were removed between steps by placing slides vertically on absorbent paper. After the final staining step, slides were gently rinsed with distilled water and dried at room temperature. Sperm morphology was subsequently evaluated under light microscopy at 400× magnification.

Hemacolor^®^ staining was performed using the commercial rapid-staining kit from Merck KGaA (Darmstadt, Germany). The kit contains three ready-to-use solutions for fixation and differential staining, along with a pH-7.2 phosphate buffer. Pre-prepared air-dried smears were sequentially immersed in the methanol fixative, the eosin-based red stain, and the azure/methylene blue, a blue stain, with gentle movement in each solution to ensure even staining. Excess reagent was removed between steps by placing slides vertically on absorbent paper. After the final staining and buffering step, slides were briefly rinsed with distilled water, dried at room temperature, and examined under light microscopy at 400× magnification.

#### 2.2.3. Spermac^®^ and Formol-Citrate Bengal Rose Staining

Spermac^®^ staining was performed using the commercial staining kit from Minitüb GmbH (Art. No. 15405/0000, Tiefenbach, Germany). This method enables clear assessment of the acrosomal status through differential coloration of the sperm head. The kit contains four solutions: a fixative and three staining reagents. Solution A stains the nucleus red, Solution B stains the equatorial segment light green, and Solution C stains the acrosome and tail dark green. The air-dried native smears were first immersed in the fixative for 5–6 min, blotted on filter paper, and dried for 15 min on a warming plate. Before and between staining steps, slides were dipped five times into distilled water to remove excess reagent. Staining was then carried out sequentially according to the manufacturer’s instructions. After the final step, slides were dried on a warming plate for approximately five minutes. Sperm evaluation was performed under light microscopy at 1000× magnification with oil immersion. Two slides were prepared to ensure unstained areas remained available for repeated assessments.

Rose Bengal is a penetrative dye that stains all sperm membranes after fixation, regardless of viability. The resulting pink coloration is a staining artifact and not a reliable marker of non-viable cells. In this study, it was used solely to enhance morphological visualization, not to assess sperm vitality. For this study, three drops of semen were mixed with 300 µL of Formol-citrate Bengal Rose solution in an Eppendorf reaction tube (Cat. No. 0030 120.086; Eppendorf SE, Hamburg, Germany). The staining solution was prepared by dissolving 2.9 g trisodium citrate dihydrate (Art. No. 106448, Merck KGaA, Darmstadt, Germany) in 100 mL distilled water, followed by the addition of 4 mL of 35% formaldehyde solution (Art. No. 252549, Merck KGaA, Darmstadt, Germany) and 0.156 g Bengal rose (Art. No. 198250, Merck KGaA, Darmstadt, Germany). Subsequently, 10 µL of the sperm suspension were pipetted onto a microscope slide and covered with a coverslip (24 × 24 mm). Prior to evaluation, the samples were allowed to rest for at least 30 min to enable the sperm cells to settle in a flattened position, ensuring accurate assessment of head morphology [26]. Microscopic evaluation was performed at 1000× magnification using oil immersion on a light microscope. Two slides were prepared per sample to ensure that, for repeat examinations, areas not previously exposed to immersion oil were available.

### 2.3. Statistical Analysis

Data handling and visualization were performed in Microsoft^®^ Excel^®^ (2024). Statistical analyses were carried out by the Working Group for Biomathematics and Data Processing, Faculty of Veterinary Medicine, Justus Liebig University Giessen, using SAS^®^ (SAS Institute Inc., version 9.4 (SAS Institute Inc., Cary, NC, USA, 2024).2024). The effects of cryopreservation, staining method, storage duration, and stress testing on sperm pathomorphology were examined across four comparisons: pre-freeze vs. post-thaw morphology, differences among staining methods at t_0_, temporal staining stability, and stress-test effects within each stain. Descriptive statistics included mean, standard deviation, range, and median value. Normality was assessed using standard tests. Depending on distribution, paired tests or non-parametric methods (Wilcoxon, Friedman) were applied, with Bonferroni correction for multiple comparisons. Ordinal staining-quality variables were analyzed using non-parametric repeated-measures tests. Statistical significance was set at *p* ≤ 0.05, with *p* ≤ 0.01 considered highly significant.

During the preparation of this manuscript, the authors used ChatGPT (GPT-4o; OpenAI, San Francisco, CA, USA, 2024) for the purposes of reference formatting. The authors have reviewed and edited the output and take full responsibility for the content of this publication.

## 3. Results

### 3.1. Time Consumption and Cost Analysis of Staining

As presented in Table 1, the economic evaluation of the staining methods considered both time requirements and material costs per slide. Substantial differences were observed between methods, particularly regarding total processing time, whereas material costs remained comparatively low across all techniques. Eosin showed the shortest total processing time (8 min and 9 s), followed by Diff-Quick^®^ (10 min and 14 s), Hemacolor^®^ (10 min and 28 s), and Eosin–Nigrosin (10 min and 32 s). In contrast, Spermac^®^ (29 min and 55 s) and Formol-citrate Bengal Rose (36 min and 0 s) required substantially longer processing times, mainly due to extended incubation or drying steps. Notably, Eosin and Eosin–Nigrosin allow additional vitality assessment, which increases evaluation time.

Material costs per slide were low for all methods, ranging from 0.13 € (Eosin) to 1.04 € (Spermac^®^). Slightly higher but still moderate costs were observed for Diff-Quick^®^ (0.46–0.91 €) and Hemacolor^®^ (0.69–2.28 €), depending on reagent consumption. Formol-citrate Bengal Rose (0.24 €) and Eosin–Nigrosin (0.26 €) also showed minimal material expenses. Overall, while material costs contributed only marginally to total expenses, processing time differed markedly between methods and represents the main factor influencing economic efficiency.

### 3.2. Staining Quality Assessment and Overall Morphology Results

Staining quality was evaluated using the parameters color intensity, detail recognition, and contrast. Qualitative observations were converted into numerical scores using a seven-point ordinal scale (1 = poor/pale, 7 = excellent/distinct). Because these variables were ordinal in nature, results are presented as median values (Md). Across all evaluation time points (t_0_–t_3_) and under stress-test conditions, all six staining methods remained suitable for evaluation. When staining quality was considered across all time points, Formol-citrate Bengal Rose staining achieved the highest overall median staining quality (Md SQ = 6). At the initial evaluation time point (t_0_), Spermac^®^ staining showed the highest staining quality (Md SQ = 7). The median values for color intensity, structural detail, contrast, and overall staining quality across all time points are summarized in Table 2. To evaluate differences between staining methods at t_0_ without stress testing, the parameters color intensity, structural detail, and contrast were analyzed statistically using the Friedman test. The analysis revealed highly significant overall differences among staining methods for all three parameters (*p* < 0.0001). After adjustment for multiple comparisons, no significant pairwise differences remained for color intensity, whereas significant differences between staining methods persisted for structural detail and contrast. In addition to staining quality, pathomorphological sperm abnormalities assessed at t_0_ without stress testing were analyzed for statistical differences between staining methods. As most variables did not follow a normal distribution, non-parametric tests were applied. Only the parameters loop-shaped tail, coiled tail, and detached head showed an approximately normal distribution and were therefore additionally described using mean values where appropriate.

Pathomorphological sperm abnormalities assessed at t_0_ without stress testing are summarized in Table 3. Only parameters with sufficient prevalence and appropriate data distribution were included in the statistical analysis. The total proportion of morphological abnormalities differed significantly between staining methods (*p* = 0.002), with median values ranging from 13.25% (Diff-Quick^®^) to 22.25% (Formol-citrate Bengal Rose). The abnormality giant head also showed a statistically significant overall difference between staining methods (*p* = 0.008), although the median proportions were low across all methods. Among tail abnormalities, loop-shaped tails showed a significant difference between staining methods (*p* = 0.003), with higher median values observed for Spermac^®^ (8.75%) and Formol-citrate Bengal Rose (8.25%) compared to the other staining methods. Rudimentary tail abnormalities likewise differed significantly between staining methods (*p* = 0.005), with median values ranging from 1% (Formol-citrate Bengal Rose) to 4% (Diff-Quick^®^). In contrast, no statistically significant differences between staining methods were observed for neck fracture (*p* = 0.108), coiled tail (*p* = 0.488), broken/bent tail (*p* = 0.454), or detached head (*p* = 0.19).

### 3.3. Eosin and Eosin–Nigrosin

**Figure 1:**
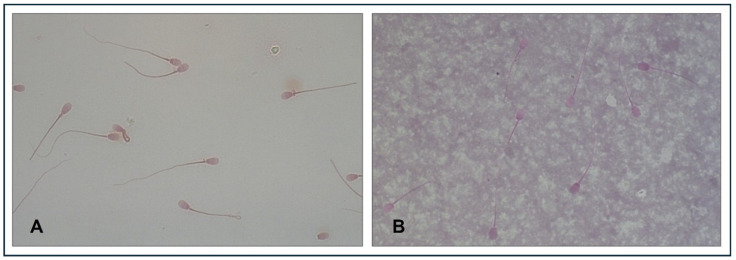
Eosin staining of thawed canine semen at 400× magnification (**A**) and Eosin–Nigrosin staining of thawed canine semen at 1000× magnification with immersion oil; heavy pigment deposition limits visibility of the tail ends (**B**).

Eosin staining showed good median values across all quality criteria and was always evaluable. Reduced visibility of sperm tails was observed in 6 out of 40 slides (Figure 1A). Occasional crystal formation, air bubbles, wavy tails, and fragmented tails (*n* = 4) were classified as artifacts. Contrast remained stable during the three-month storage period (median = 5; Table 2), while color intensity and detail recognition also maintained median values of 5 with minor fluctuations. At t_0_, detail recognition differed significantly between Eosin and Spermac^®^ and between Eosin and Formol-citrate Bengal Rose (*p* = 0.029). Eosin-stained slides showed the highest proportion of broken or bent tails (median = 1.5%), and the overall median proportion of pathomorphological sperm was 18.75%. Although total pathology differed across staining methods at t_0_ (*p* = 0.002), no pairwise differences remained after Bonferroni correction.

All Eosin–Nigrosin slides (*n* = 80) were evaluable at all time points. Color intensity remained high (median = 5), whereas overall staining quality was lowest among methods (median = 3) and declined slightly over time, making pathomorphological assessment at 1000× magnification more time-consuming in some slides. Frequently observed features included high pigment (*n* = 11; Figure 1B), limited tail visibility (*n* = 30), and poor tail visibility (*n* = 36). At t_0_, significant differences were found in detail recognition compared with Spermac^®^ and Formol-citrate Bengal Rose, and in contrast compared with Diff-Quick^®^, Spermac^®^, and Formol-citrate Bengal Rose (*p* = 0.029). The median proportion of sperm with rudimentary tails was 3.65%, significantly higher than with Formol-citrate Bengal Rose (0.85%, *p* = 0.029). The median proportion of all pathomorphological sperm was 16.25%, the second lowest among staining methods.

### 3.4. Diff-Quick^®^

**Figure 2:**
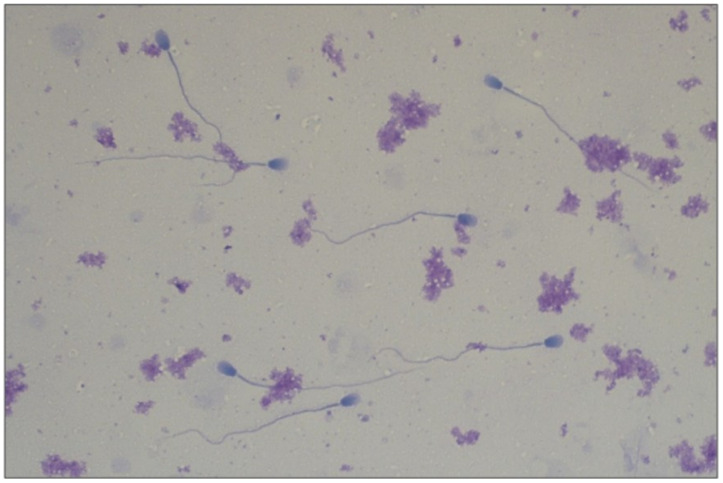
Diff-Quick^®^ staining of thawed canine semen at 400× magnification with wavy tails and pigment clusters.

All 40 Diff-Quick^®^ slides were evaluable at all time points. Median values for color intensity, detail recognition, contrast, and overall staining quality were almost exclusively 5, except for contrast at t_0_, which was slightly higher at 6 (Table 2). Statistical analysis at t_0_ without stress testing revealed a significant difference in contrast compared to Eosin–Nigrosin (*p* = 0.029). Pathomorphological assessment revealed significant differences in loop-shaped sperm tails, with a median of 5.5% for Diff-Quick^®^ versus 8.75% for Spermac^®^ (*p* = 0.029, Bonferroni-corrected). Detached heads were identified as least frequent in this stain (median 0.5%). Diff-Quick^®^ slides had the highest median for rudimentary tails at 4% (Table 3). Wavy tails and pigment nests were observed in 15 and 14 slides, respectively (Figure 2). Overall, the median proportion of pathomorphological sperm was lowest for Diff-Quick^®^ at 13.75%.

### 3.5. Hemacolor^®^

**Figure 3:**
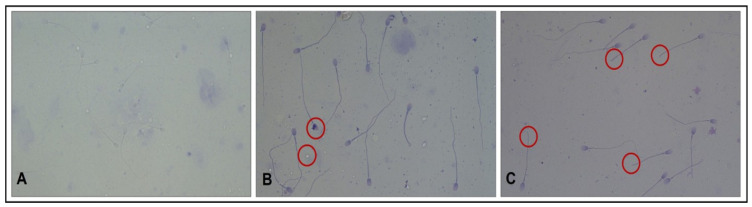
Hemacolor^®^ staining of thawed canine semen at 400× magnification shows (**A**) pale staining with pigment clusters, wavy tails, poorly visible tail ends, and droplet formation, (**B**) wavy tail ends with droplet formation and stain particles, and (**C**) stain particles with poorly discernible tail ends (marked).

All 40 Hemacolor^®^ slides were evaluable at all four time points. Median quality scores were consistently 5 across all evaluations, with minor deviations within ±0.5 at single time points. Color intensity and contrast showed slight fluctuations between 4 and 5 over storage, while detail recognition slightly decreased to 4.5 at t_3_ (Table 2). Some slides showed strong fluctuating color intensity during evaluation and appeared pale (Figure 3A). Poorly discernible sperm tail ends were observed in several Hemacolor^®^-stained slides (*n* = 12, Figure 3B). Pigment accumulations (*n* = 9, Figure 3C) as well as wavy tail ends (*n* = 10) were also frequently noted. Hemacolor^®^ slides showed the lowest median proportion of loop-shaped tails (5%) and a median of 17.75% for overall pathomorphological changes, placing it in the mid-range among all staining methods.

### 3.6. Spermac^®^

**Figure 4:**
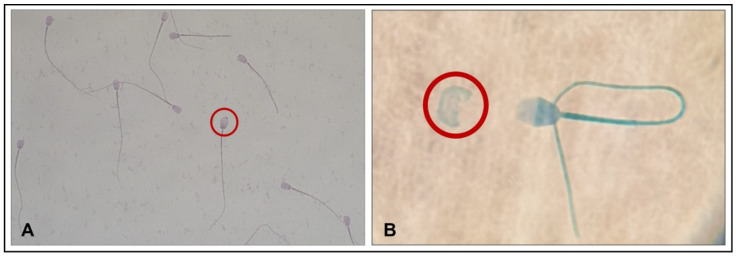
Spermac^®^ staining of thawed canine semen at 1000× magnification with immersion oil. (**A**) giant head and (**B**) cropped section showing detached acrosomal cap (marked).

All 80 Spermac^®^ slides were evaluable at all four times. At t_0_, detail recognition and contrast were highest among all methods (medians 7 and 6.5, respectively), with overall staining quality also highest (median 7, Table 2). Detail recognition remained superior throughout storage (median 6), though median values declined to 4–5 by t_3_. Statistical analysis at t_0_ without stress testing showed significant differences in detail recognition and contrast compared to Eosin and Eosin–Nigrosin (*p* = 0.029). Pathological features with increased prevalence included detached acrosomes (median 0.25%) and loop-shaped tails (median 8.75%, mean 10.05%), the latter significantly higher than Diff-Quick^®^ (*p* = 0.029). The median proportion of pathomorphological sperm was 20.25%, the second highest among all methods. Other notable findings were pigment clusters (*n* = 8) and occasional giant heads (Figure 4A), with some slides showing pigment loss at t_3_ (Figure 4B). Overall, Spermac^®^ slides had the fewest total anomalies (*n* = 19).

### 3.7. Formol-Citrate Bengal Rose

All 80 slides were evaluable at all four time points. Median overall staining quality was highest at 6 throughout the storage period, with color intensity and contrast medians also at 6. No median fell below 5 at any time point. Over three months, medians declined slightly by 0.5–1 point, less than the decline observed for Spermac^®^ (Table 2).

**Figure 5:**
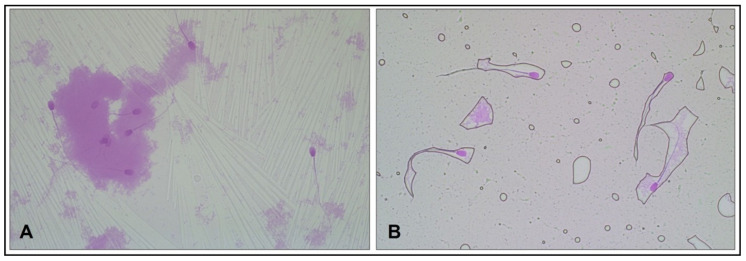
Formol-citrate Bengal Rose staining of thawed canine semen at 1000× magnification with immersion oil shows pronounced dye crystal formation with sperm pigment clusters at t_3_ (**A**) and air inclusions surrounding pigment-rich sperm cells (**B**).

Statistical analysis at t_0_ without stress testing revealed significant differences in detail recognition compared to Eosin and Eosin–Nigrosin (*p* = 0.029 each) and significant contrast differences compared to Eosin–Nigrosin (*p* = 0.029). Formol-citrate Bengal Rose showed the highest median proportion of overall pathomorphological abnormalities at 22.25%. Rudimentary tails were rare (median 1%) compared to Eosin–Nigrosin (median 3.25%, *p* = 0.029) and broken/bent tails were least frequent (median 0.25%). Loop-shaped tails were relatively common (median 8.25%, mean 11.7%, Table 3). Lance-shaped sperm heads were notable, with 5.2% of sperm at t_0_ showing this morphology (median 2.25%), while other stains had a median of 0%. Other anomalies included air bubbles with clustered sperm (*n* = 13), pigment clusters (*n* = 8), and, at t_3_ strong crystal formation (*n* = 3, Figure 5 A,B).

### 3.8. Comparison of Staining Methods with and Without Stress Testing

Finally, at t_0_ without stress testing, the six staining methods showed median prevalence rates of morphological abnormalities ranging from 13.25% to 22.25% of the analyzed spermatozoa. The initial statistical analysis revealed a highly significant difference between the staining methods (*p* = 0.0018). However, after *p*-value adjustment, no statistically significant pairwise comparisons could be identified. The statistical evaluation of the parameter “giant heads” likewise revealed a significant difference between the staining methods in the initial analysis (*p* = 0.008). However, after adjustment for multiple testing, no significant pairwise comparisons remained (Table 3). The most frequently observed pathology was looped tails, with a median prevalence of 5–8.75%. A significant difference between the six staining methods was detected (*p* = 0.0027). After Bonferroni correction, one pairwise comparison remained significant, showing a difference between Diff-Quick^®^ and Spermac^®^ staining (*p* = 0.0293). A detailed presentation of the prevalence distributions, including minima, maxima, and quartiles, is provided for this most prevalent abnormality in Table 4. The second most common morphological abnormality was rudimentary tail structures, with median values ranging from 1 to 4% (*p* = 0.0045). After multiple pairwise comparisons, a significant difference remained between Eosin–Nigrosin and Formol-citrate Bengal Rose staining (*p* = 0.0293).

Furthermore, pre-staining storage temperature affected canine sperm morphology, while staining quality remained stable (Table 5). Rare abnormalities (acrosomal defects, vacuoles, distal droplets) were too infrequent for analysis, so statistics focused on the high-prevalence features, looped tails, rudimentary tails, and detached heads, alongside color intensity, detail recognition, and contrast. Stress-exposed samples, particularly at 37 °C, showed higher median proportions of pathomorphological sperm (19.3–21.6%) than unstressed controls (17.8%), with rudimentary tails also increased (3.5–4.0% vs. 2.8%; *p* ≤ 0.035). Across staining methods, trends were consistent: Eosin, Eosin–Nigrosin and Diff-Quick^®^ exhibited the highest total pathologies at 37 °C; Eosin and Spermac^®^ showed significant increases in rudimentary tails under stress; Hemacolor^®^ had minor, non-significant increases; Formol-citrate Bengal Rose showed no significant differences, though lance-shaped sperm heads were noted only in this stain. “Neck break” medians peaked in Eosin–Nigrosin and Hemacolor^®^ after 6 °C stress (1.5%).

Storage effects on unstressed canine sperm were assessed across all staining methods over four time points (t_0_–t_3_) (Table 6). Analysis focused on high prevalence pathomorphological features (neck break, looped tails, rolled tails, rudimentary tails) and staining quality parameters (color intensity, detail recognition, contrast). Due to non-normal distribution, Friedman tests with Bonferroni correction were applied (*p* ≤ 0.05). Overall, total pathomorphology remained largely stable during storage, with only minor fluctuations between time points. Eosin and Eosin–Nigrosin showed slight decreases in color intensity and contrast at t_3_, while Diff-Quick^®^ and Hemacolor^®^ exhibited small, non-systematic variations in individual defects. The staining quality of Formol-citrate Bengal Rose remained stable for color intensity across all time points, with slight decreases for detail recognition and contrast at t_3_. In comparison, Spermac^®^ demonstrated significant declines in color intensity, detail recognition, and contrast over time. Lance-shaped sperm heads observed in Formol-citrate Bengal Rose were observed only at t_0_, whereas the median was 0% at all subsequent time points. In summary, storage duration had minimal impact on sperm morphology, and staining quality was largely preserved, except for a decline in quality parameters in Spermac^®^ over time.

### 3.9. Effect of Cryopreservation on Sperm Pathomorphology

To assess the impact of cryopreservation on sperm morphology, the baseline examination before freezing (t − 1, Eosin staining) was compared with the post-thaw condition at t_0_ across all staining methods. Normality was evaluated using Shapiro–Wilk, Kolmogorov–Smirnov, Cramér–von-Mises, and Anderson–Darling tests. Depending on distribution, paired *t*-tests or Wilcoxon signed-rank tests were applied. Loop-shaped tails increased significantly after cryopreservation in all staining methods except Hemacolor^®^, with post-thaw values at least twice as high as at t − 1 (mean 2.4% ± 1.94). The highest prevalence was observed in Formol-citrate Bengal Rose (11.7%, *p* = 0.014). In contrast, the proportion of broken or bent tails decreased significantly in five of six staining methods, with lower post-thaw values compared to the baseline mean of 4.2% ± 3.54. Detached heads were less frequent after thawing than at t − 1 (2.75% ± 2.76), with significant reductions in Eosin and Eosin–Nigrosin (*p* = 0.039 and *p* = 0.047). No significant differences were detected for the remaining staining methods. Detailed results are presented in Table 7.

## 4. Discussion

In canine reproductive medicine, artificial insemination with cryopreserved semen has gained increasing importance in recent years [44,45]. Within this context, rigorous semen quality control is indispensable prior to insemination or cryopreservation. In addition to vitality, sperm concentration, and microbiological status, the morphological integrity of spermatozoa represents a decisive determinant of fertilization success. Earlier investigations [1,10] already emphasized the prognostic value of morphology and demonstrated a clear negative correlation between increasing proportions of abnormal spermatozoa and fertilizing capacity. Nevertheless, the literature reveals variability in the definition of acceptable limits for pathological forms in dogs. In this context, twenty to thirty percent morphologically abnormal spermatozoa are considered within the normal range [3], while a maximum of 20% has also been proposed [24], and values of 10–25% are described as physiologically acceptable [2]. Despite these differences, there is general agreement that increased morphological abnormalities may reduce conception rates. Accordingly, sperm morphology is regarded as one of the three key parameters in canine semen evaluation, together with total sperm count and sperm viability [4].

Because unstained semen samples provide insufficient contrast under light microscopy and limit visualization of fine structural details, staining techniques are routinely applied to enhance morphological evaluation. The present study therefore aimed to compare several established staining methods regarding their suitability for assessing canine sperm morphology, particularly in frozen–thawed semen. To strengthen statistical power and provoke a higher prevalence of pathological forms, additional stress tests at different post-thaw storage temperatures were conducted. Beyond diagnostic performance, practical aspects such as preparation time, material costs, and long-term archiving stability were examined to provide clinically relevant recommendations for veterinary practice. More complex fluorescence or nucleic acid-based methods were deliberately excluded, as these require specialized equipment and are primarily used in research settings. Although some cited primary studies are older, the staining protocols themselves have remained methodologically stable over decades. While the historical development of sperm staining across mammalian species is well documented, contemporary data focusing specifically on canine pathomorphology remain limited. Likewise, systematic analyses of stress-induced morphological changes, storage stability, and economic considerations are scarce, underscoring the relevance of the present investigation.

Methodologically, semen samples from ten proven fertile breeding dogs of eight different breeds were included to reflect realistic clinical diversity. All samples were processed using the established Uppsala Equex-2 system as diluent, and two 0.5 mL straws per dog were thawed according to World Health Organization [40] recommendations. Even though frozen–thawed semen is still less commonly used than fresh or chilled semen, its clinical relevance is increasing due to the possibility of utilizing genetically valuable stud dogs independent of time and location [46]. Because cryopreserved samples were expected to show relatively low baseline abnormality rates, additional stress tests were performed by incubating aliquots for two hours at 6 °C, 18 °C, and 37 °C, while a fourth fraction served as control. Previous studies [47,48,49] suggest that stress conditions may increase the occurrence of morphological defects, thereby facilitating comparative analyses. However, it is well established that staining procedures can induce artifacts or structural alterations [50,51], and that additional mechanical distortions may arise during smear preparation. In the present study, lance-shaped sperm heads were predominantly observed at t_0_ in the Formol-citrate Bengal Rose stain but decreased markedly in subsequent evaluations, indicating a likely preparation-related artifact caused by insufficient flattening time. Although a spreading time of approximately 30 min has been recommended in the literature [29], the present observations suggest that longer incubation periods may be required for thawed canine semen.

Morphological evaluation followed established criteria [3]. For each sample, 200 spermatozoa were analyzed using an electronic counter. While this number meets commonly accepted standards, it still represents a relatively limited sample size. In accordance with the approach described in [52], only the most prominent defect per spermatozoon was recorded. This method simplifies classification but may underestimate the presence of multiple concurrent abnormalities. To minimize inter-observer variability, all assessments were performed by a single examiner, as recommended in [53]. Although this strategy enhances consistency, it may also reduce the overall reproducibility of the findings. Economic assessment incorporated preparation and evaluation time calculated using an hourly wage of €12.69, as well as material costs, while excluding fixed laboratory infrastructure. Although reflective of practical conditions, these calculations are subject to price fluctuations as of 28 October 2024. Archiving stability was examined over three months, with most preparations mounted under coverslips and stored dry and protected from light, except for Formol-citrate Bengal Rose, which remained a wet preparation.

Regarding economic expenditure, preparation and evaluation times varied considerably between methods. Eosin required the shortest total time, whereas Formol-citrate Bengal Rose was most time-consuming due to the flattening period, although omission of this step would substantially reduce preparation time. Spermac^®^ also demanded considerable time because of multiple staining steps. Evaluation at 400× magnification facilitated faster counting, whereas 1000× oil immersion improved detail recognition at the expense of time. Material costs ranged from very low for non-commercial stains such as Eosin, Eosin–Nigrosin, and Formol-citrate Bengal Rose to substantially higher costs for commercial systems such as Hemacolor^®^, particularly with increased reagent consumption. When labor was included, Eosin proved the most economical overall, while Formol-citrate Bengal Rose was most expensive unless waiting times were efficiently integrated into workflow. When comparing the applied staining methods, it became evident that each technique fulfilled the basic requirements for morphological assessment of frozen–thawed canine semen yet differed in diagnostic detail and practical handling. Formol-citrate Bengal Rose provided the most differentiated visualization and remained stable over time despite being stored as a wet preparation and requiring careful preparation. Spermac^®^ allowed precise acrosome evaluation with the highest rated staining quality at t_0_ but demonstrated reduced color intensity during prolonged storage. Diff-Quick^®^ and Hemacolor^®^ yielded consistently interpretable results with only minor methodological limitations. However, depending on staining quality, looped tails may potentially be misclassified as rudimentary tail ends, which could influence the reported distribution of abnormalities. Notably, differences in the prevalence of morphological abnormalities between staining methods were observed. The comparatively high prevalence detected with Formol-citrate Bengal Rose at t_0_ is likely attributable to the frequent occurrence of lance-shaped sperm heads, which were identified as preparation-related artifacts rather than true biological abnormalities. In contrast, Diff-Quick^®^ tended to yield lower abnormality rates, which may reflect reduced detection sensitivity for subtle structural defects or a closer approximation to the true physiological condition. These findings suggest that variations between staining methods are influenced not only by biological factors but also by method-specific visualization properties and artifact susceptibility. In contrast, Eosin and Eosin–Nigrosin stood out primarily for their efficiency and low cost, while offering comparatively less detailed structural resolution. Although variations in contrast and detail recognition were measurable, many differences in overall pathological rates were no longer statistically significant after adjustment for multiple testing.

Cryopreservation significantly increased the proportion of looped tails and overall morphological abnormalities, consistent with known cryo-induced structural damage [45,54]. The observed reductions in bent tails and detached heads likely reflect methodological or classification-related effects rather than true biological improvement. Additional stress testing at 37 °C further increased total abnormalities and rudimentary tails, in line with previous findings [47,55], and these effects are plausibly attributable to temperature-induced membrane and cytoskeletal damage [56,57]. In contrast, cooling stress at lower temperatures produces less consistent changes. Importantly, staining quality parameters remained stable across all stress conditions, indicating that the observed differences were biologically induced rather than artifactual. Regarding storage stability, most staining methods maintained consistent morphological visualization over a three-month period, in agreement with WHO recommendations [35].

## 5. Conclusions

This study shows that established staining techniques remain reliable for evaluating the morphology of cryopreserved canine semen. Because sperm shape strongly influences fertilization alongside count and viability, accurate assessment is essential for quality control. All six stains were usable, though they differed in quality, time, cost, and stability. Formol-citrate Bengal Rose produced the strongest coloration and detail but required precise, time-intensive handling. Spermac^®^ offered the best acrosome visualization but faded during storage. Diff-Quick^®^ and Hemacolor^®^ gave consistent results, while Eosin and Eosin–Nigrosin were the fastest and most economical; Eosin was the most cost-effective overall. Cryopreservation increased abnormalities, especially looped tails, and incubation at 37 °C further raised defect rates, confirming thermal and cryo-induced damage. Staining quality remained stable under stress, indicating biological rather than methodological changes. All slides stayed evaluable for three months. Despite limitations, the findings reflect routine laboratory conditions and confirm that conventional stains remain robust, practical tools for frozen–thawed semen evaluation.

## Figures and Tables

**Table 1:** Cost analysis of staining methods per slide ranked by ascending total costs (€).

Staining Method	Material Components	Materials (€)	TT (min:s)	TT (min)	LC (€)	TC (€)
Eosin	0.02 mL Eosin solution, 2 slides, 2 pipette tips	0.13	8:15	8.15	1.72	1.85
Eosin–Nigrosin	0.03 mL Eosin–Nigrosin solution, 2 slides, 3 pipette tips, 0.05 mL immersion oil	0.26	10:54	10.54	2.23	2.49
Diff-Quick^®^	3 × 2–5 mL staining solution, 2 slides, 1 pipette tip, 50 mL aqua dest.	0.46–0.91	10:24	10.24	2.17	2.63–3.08
Hemacolor^®^	4 × 0.5–2 mL staining solution, 2 slides, 1 pipette tip, 50 mL aqua dest.	0.69–2.28	10:46	10.46	2.21	2.90–4.49
Spermac^®^	4 × 0.25 mL staining solution, 2 slides, 1 pipette tip, 0.05 mL immersion oil, 50 mL aqua dest.	1.04	29:91	29.91	6.33	7.37
Formol-citrate Bengal Rose	0.3 mL stain solution, 1 slide, 1 coverslip, 2 pipette tips, 0.05 mL immersion oil, 1 coverslip	0.24	36:00	36.00	7.61	7.85

Note: TT: Total time; Min: Minute; S: Second; LC: Labor cost; TC: Total cost. Each entry represents one stained slide (200 sperm cells). Costs include reagents, slides, coverslips, pipette tips, aqua dest. and immersion oil. Total time combines preparation, incubation/drying, and microscopic evaluation. Labor costs were calculated based on 0.2115 € per minute. Total costs include labor and material costs per slide.

**Table 2:** Median staining quality of the six staining methods across evaluation time points (t_0_–t_3_).

Staining Method	Parameter	Median (t_0_–t_3_)	t_0_	t_1_	t_2_	t_3_
Formol-citrate Bengal Rose	Color intensity (1–7)	6	6.5	6	6	6
	Detail recognition (1–7)	5	6	6	5	5
	Contrast (1–7)	6	6	6	5	5
	Overall staining quality (Md SQ)	6	6	6	6	5
Spermac^®^	Color intensity (1–7)	5	6	6	5	4
	Detail recognition (1–7)	6	7	6	5	5
	Contrast (1–7)	5	6.5	6	5	4
	Overall staining quality (Md SQ)	5	7	6	5	5
Diff-Quick^®^	Color intensity (1–7)	5	5	5	5	5
	Detail recognition (1–7)	5	5	5	5	5
	Contrast (1–7)	5	6	5	5	5
	Overall staining quality (Md SQ)	5	5	5	5	5
Eosin	Color intensity (1–7)	5	5	5	5	5
	Detail recognition (1–7)	5	5	5	5	5
	Contrast (1–7)	5	5	5	5	5
	Overall staining quality (Md SQ)	5	5	5	5	5
Hemacolor^®^	Color intensity (1–7)	5	5	4.5	5	4
	Detail recognition (1–7)	5	5	5	5	4.5
	Contrast (1–7)	5	5	4	4.5	4
	Overall staining quality (Md SQ)	5	5	5	5	4
Eosin–Nigrosin	Color intensity (1–7)	5	5	5	5	4
	Detail recognition (1–7)	3	4	3	3	3
	Contrast (1–7)	3	4	3	3	3
	Overall staining quality (Md SQ)	3	5	3	3	3

Note: Data were organized by sample and slide and evaluated across the four-time points t0–t3, corresponding to after preparation (t0), after 24 h (t1), after 7 days (t2), and after 3 months (t3). 200 cells per slide were assessed using a light microscope. Results are reported as medians. Statistical analysis was performed using non-parametric methods, including the Friedman test for repeated measures, with adjustment for multiple comparisons where applicable (*p* ≤ 0.05 considered significant). Color intensity, detail recognition, and contrast were rated on a seven-point ordinal scale (1 = poor/pale, 7 = excellent/distinct). Median overall staining quality (Md SQ) represents the combined evaluation of these parameters.

**Table 3:** Median proportions (%) of pathomorphological sperm abnormalities per staining method at t_0_ without stress testing.

Pathomorphological Feature	E	EN	DQ	H	S	FB	MD-Value	*p*-Value
Total morphological abnormalities	18.75	16.25	13.25	17.75	20.25	22.25	19.04	0.002
Giant head	0.5	0.5	0	0	0.75	0	0.375	0.008
Neck fracture	1	1	0.75	0.25	0	1	0.625	0.108
Loop-shaped tail	5.25	6.75	5.5	5	8.75	8.25	6.25	0.003
Coiled tail	0.75	0	0.5	0.25	1	0.5	0.5	0.488
Broken/bent tail	1.5	0.5	1	1.25	1	0.25	1	0.454
Rudimentary tail	3	3.25	4	3	2.5	1	2.875	0.005
Detached head	1	1	0.5	1	1.5	1	1	0.19

Note: E = Eosin; EN = Eosin–Nigrosin; DQ = Diff-Quick®; H = Hemacolor®; S = Spermac®; FB = Formol-citrate Bengal Rose; Md = median (%). *p*-values were evaluated using pairwise comparisons with Bonferroni correction; no additional significance markings are applied in this table.

**Table 4:** Percent prevalence of looped spermatozoa at t_0_ in unstressed samples across different staining methods.

Staining Method	Median	Min.–Max.	25%	75%	90%
Eosin	5.25	0–16	2.5	9	13.75
Eosin–Nigrosin	6.75	1–12.5	3.5	11.5	12.5
Diff-Quick^®^	5.5	2.5–11	3.5	7.5	9.5
Hemacolor^®^	5	0–10.5	1.5	7.5	10
Spermac^®^	8.75	3.5–26	8	9.5	18.5
Formol-citrate Bengal Rose	8.25	2–30.5	6.5	16	24.75

Note: Min.–Max.: Minimum–Maximum. The table shows the prevalence of loop-shaped sperm cells at t_0_ without stress testing across the different staining methods. Statistical differences between staining methods (*p* ≤ 0.05) are indicated according to the *p*-values shown; no additional formatting is applied.

**Table 5:** Median pathomorphology and quality parameters of canine sperm at t_0_ with and without stress test.

Staining Method	Parameter	Unstressed	37 °C	18 °C	6 °C	*p*-Value
All Methods	Total pathologies (%)	17.79	21.63	19.33	20.13	0.036
	Looped tails (%)	7.46	9.13	8	7.38	0.246
	Rudimentary tails (%)	2.75	4	3.67	3.46	0.022
	Detached head (%)	1.08	0.79	1.29	1.08	0.777
	Color intensity (1–7)	5.67	5.42	5.5	5.58	0.229
	Detail recognition (1–7)	5.25	5.08	5.08	5.25	0.871
	Contrast (1–7)	5.67	5.25	5.17	5.33	0.265
Eosin	Rudimentary tails (%)	3	8	6.75	4	0.032
Eosin–Nigrosin	Total pathologies (%)	16.25	22.5	16.25	19	0.007
Diff-Quick^®^	Total pathologies (%)	13.25	20.25	15.25	18.25	0.042
Hemacolor^®^	Total pathologies (%)	17.75	17.5	18.75	20.75	0.106
Spermac^®^	Rudimentary tails (%)	2.5	1.75	3.5	3.25	0.011
Formol-citrate Bengal Rose	Total pathologies (%)	22.25	20.75	19.75	24.5	0.669

Note: Only parameters with sufficiently high prevalence were included; rare abnormalities were excluded. Statistical differences between temperature conditions (*p* ≤ 0.05) are reported based on the corresponding *p*-values; no additional significance formatting is applied.

**Table 6:** Median pathomorphology (%) and quality parameters (1–7) across storage times (t_0_–t_3_) by staining method (unstressed).

	Time	E	EN	DQ	H	S	FB
Total pathologies (%)	t_0_	18.75	16.25	13.25	17.75	20.25	22.25
	t_1_	15.25	17.5	14.5	16.75	19.25	18
	t_2_	15.25	14	13.75	11.5	21	16.5
	t_3_	11.5	10.75	13	12.25	26	18.5
Neck break (%)	t_0_	1	1	0.75	0.25	0	1
	t_1_	0.5	1.5	1	1.25	1	1.25
	t_2_	0.25	1.25	0.25	0.75	0.75	1.25
	t_3_	0.5	0.25	1.75	0.75	1.5	1
Looped tails (%)	t_0_	5.25	6.75	5.5	5	8.75	8.25
	t_1_	4.75	7.5	4.75	5.75	7.5	8.5
	t_2_	7	5.75	5	4.5	10.5	6.75
	t_3_	4.25	4	6	5.25	8.25	10
Rolled tails (%)	t_0_	0.75	0	0.5	0.25	1	0.5
	t_1_	0.5	0.75	1.5	1	1	1.5
	t_2_	0.25	0.25	0	1	1.5	1.25
	t_3_	0.5	0.75	0.5	1	1	1.75
Rudimentary tails (%)	t_0_	3	3.25	4	3	2.5	1
	t_1_	2.25	0.75	2.25	2.5	3.5	0.5
	t_2_	1.5	1.25	2	1.5	1.5	1.5
	t_3_	2	1.25	2.75	3.75	2.5	1
Color intensity (1–7)	t_0_	5	5	5.5	5	7	6
	t_1_	5	5	5	4	5	6
	t_2_	5	5	5.5	4.5	4	6
	t_3_	4.5	4.5	5	4	3.5	6
Detail recognition (1–7)	t_0_	4.5	3.5	5	5	7	6
	t_1_	5	3	5	5	6	6
	t_2_	5	3	5	5	5	5.5
	t_3_	5	3	5	4.5	5	5
Contrast (1–7)	t_0_	5	3.5	6.5	5.5	7	6
	t_1_	5	3	5	4.5	5	6
	t_2_	5	3	5.5	5	5	5
	t_3_	4.5	3	5	3.5	4	5

Note: E = Eosin; EN = Eosin–Nigrosin; DQ = Diff-Quick®; H = Hemacolor®; S = Spermac®; FB = Formol-citrate Bengal Rose.

**Table 7:** Comparison of sperm pathomorphological features before cryopreservation (t − 1, Eosin staining) and after thawing (t_0_) across all staining methods without stress testing (mean proportions in %).

Feature	Eosin t − 1	E t_0_	EN t_0_	DQ t_0_	H t_0_	S t_0_	FB t_0_
Loop-shaped tail (%)	2.4	6.2	7.15	5.6	4.95	10.05	11.7
*p*-value	—	0.033	0.022	0.029	0.075	0.004	0.014
Broken/bent tail (%)	4.2	1.35	1.0	1.35	1.5	1.15	0.55
*p*-value	—	0.038	0.036	0.040	0.089	0.026	0.016
Detached head (%)	2.75	1.0	0.95	0.85	1.35	1.75	1.15
*p*-value	—	0.039	0.047	0.095	0.168	0.578	0.069

Note: E = Eosin; EN = Eosin–Nigrosin; DQ = Diff-Quick®; H = Hemacolor®; S = Spermac®; FB = Formol-citrate Bengal Rose. Statistical differences between t − 1 and t_0_ (*p* ≤ 0.05) are reported based on the corresponding *p*-values; no additional significance formatting is applied.

## Data Availability

The datasets generated and analyzed during the current study are not publicly available due to ethical and legal restrictions related to animal data protection and institutional requirements. The data include information that could potentially compromise animal welfare oversight or institutional compliance. Data are available from the corresponding author upon reasonable request.
